# Supplementary material for: Thermosensation in Caenorhabditis elegans is linked to ubiquitin-dependent protein turnover via insulin and calcineurin signalling
Source: Nat Commun. 2022 Oct 5;13:5874. doi: 10.1038/s41467-022-33467-7 (PMC9534930; doi:10.1038/s41467-022-33467-7)
Supplement: Supplementary file 1 — Supplementary Information [file 41467_2022_33467_MOESM1_ESM.pdf]

Supplementary Information

Thermosensation in *Caenorhabditis elegans* is linked to ubiquitin-dependent protein turnover via insulin and calcineurin signalling

Segref *et al.*

Contents

Supplementary Figures 1-7

Supplementary Tables 1-6

Supplementary References

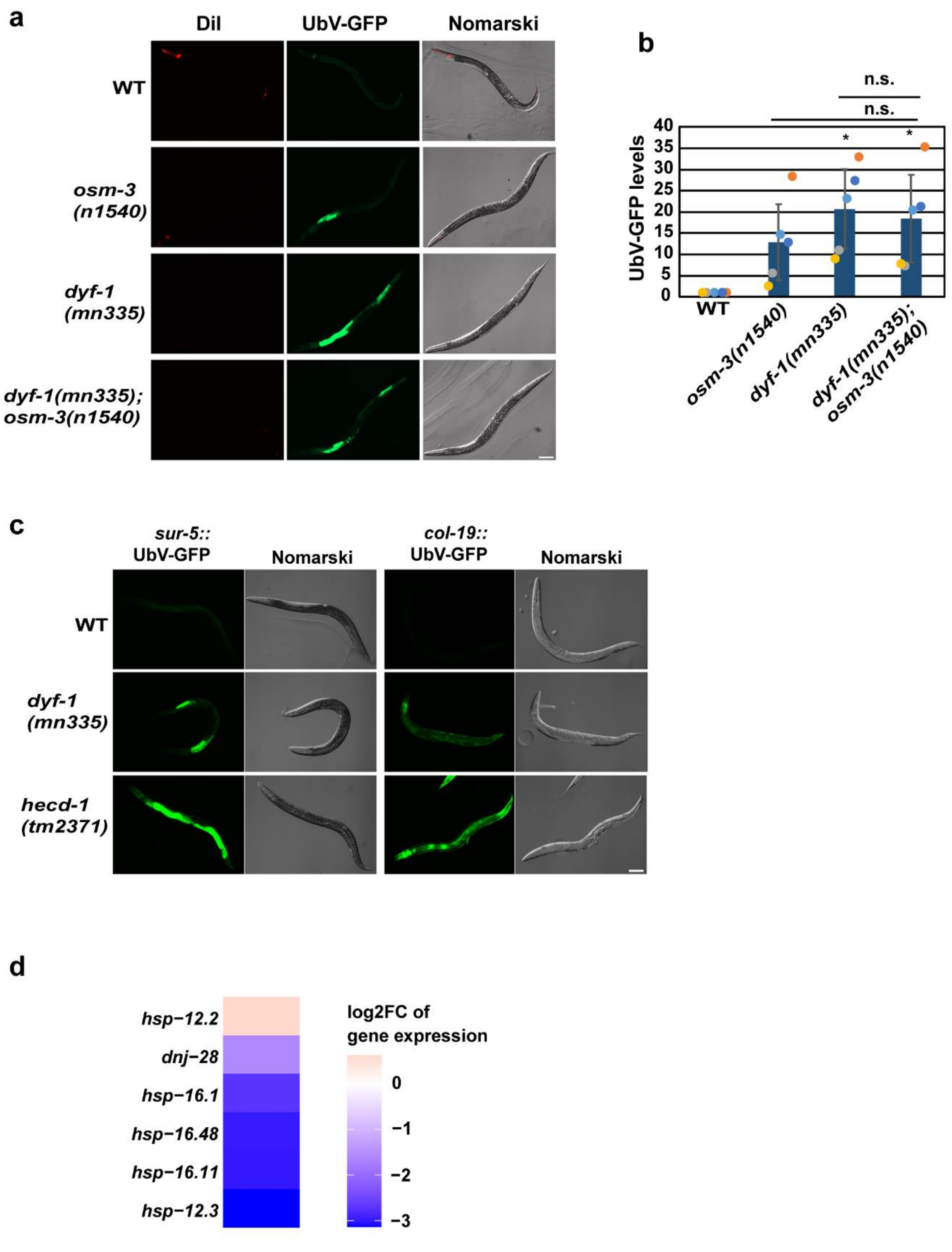

Supplementary Figure 1. UFD degradation is affected in ciliary mutants. **a** Fluorescence and Nomarski images of worms expressing the UFD substrate (UbV-GFP) in wild-type, *osm-3(n1540)*, *dyf-1(mn335),* or *dyf-1(mn335); osm-3(n1540)* mutant worms as indicated. DiI denotes ciliated amphid and phasmid neurons. n = 3 biologically independent experiments.

Scale bar: 100 μm. **b** Quantification of Western blots displayed in Figure 1c. n = 5 biologically independent experiments, WT compared to: *osm-3(n1540) p* = 0.1444, *dyf-1(mn335) p* = 0.0106, *dyf-1(mn335); osm-3(n1540) p* = 0.0232, *osm-3(n1540)* versus *dyf-1(mn335); osm-3(n1540) p* = 0.629, *dyf-1(mn335)* versus *dyf-1(mn335); osm-3(n1540) p* = 0.9229, one-way ANOVA followed by Dunnett's multiple comparisons test. Column graphs: mean +/- standard deviation, scatter plots, individual experiments, same colour: same experiment. * $p \leq 0.05$, n.s. $p > 0.05$. **c** Fluorescence and Nomarski images of worms expressing *sur-5*::UbV-GFP or *col-19*::UbV-GFP in wild-type, *dyf-1(mn335)* or *hecd-1(tm2371)* mutant worms. n = 2 biologically independent experiments. Scale bar: 100 μm. **d** Heatmap of differentially expressed chaperones (q-value ≤ 0.01) in *dyf-1(mn335)* compared to wild-type based on microarray analysis. Colour of cells: log2 fold change of gene expression in *dyf-1(mn335)* compared to wild-type, n = 4 biologically independent experiments. Source data are provided as a Source Data file.

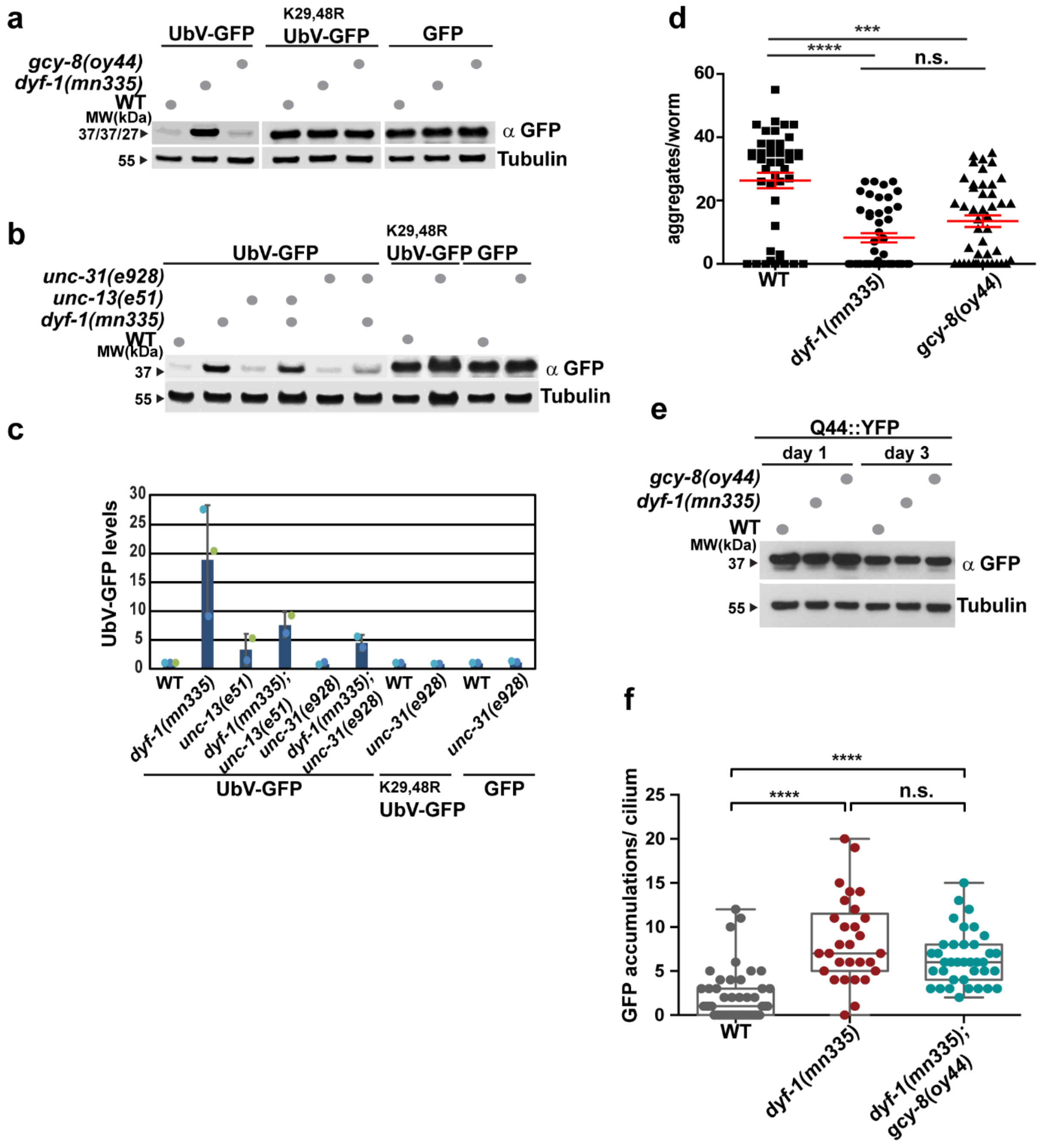

Supplementary Figure 2. Neurosecretion affects UFD degradation.

a Lysates of day 1 adult worms expressing UbV-GFP, ^{K29,48R}UbV-GFP, or GFP and the mutations indicated by a dot were analysed by Western blotting against GFP or tubulin, n = 2 biologically independent experiments. **b** Indicated mutant worms expressing UbV-GFP, ^{K29,48R}UbV-GFP or GFP alone were analysed at day 1 of adulthood by Western blotting as in (a). **c** Quantification of 2 biologically independent experiments of indicated wild-type (WT) or mutant worms. Column graphs: mean +/- standard deviation, scatter plots, individual experiments, same colour: same

experiment. **d** Quantification of the number of Q44::YFP aggregates per worm in day 3 of adulthood. Red: mean +/- standard error of the mean, WT versus: *dyf-1(mn335) p* < 0.0001, *gcy-8(oy44) p* = 0.0002, *dyf-1(mn335)* versus *gcy-8(oy44) p* = 0.1314, Kruskal-Wallis one-way ANOVA with Dunn's multiple comparisons test. **** p ≤ 0.0001, *** p ≤ 0.001, n.s. p > 0.05. n = 2 biologically independent experiments. **e** Lysates of day 1 and day 3 adult worms with the genotypes indicated by a dot expressing Q44::YFP in intestinal cells were analysed by Western blotting against GFP and tubulin, n = 4 biologically independent experiments. **f** Quantification of the number of GFP accumulations per AWA cilium analysed by ODR-10::GFP expression in the indicated wild-type (WT) and mutant strains. Boxplot of median values with 25th and 75th percentile and min. to max. whiskers are displayed. WT versus: *dyf-1(mn335) p* < 0.0001, *dyf-1(mn335); gcy-8(oy44) p* < 0.0001, *dyf-1(mn335)* versus *dyf-1(mn335); gcy-8(oy44) p* = 0.7306, Kruskal-Wallis one-way ANOVA with Dunn's multiple comparisons test. **** p ≤ 0.0001, n.s. p > 0.05, n = 2 biologically independent experiments. Source data are provided as a Source Data file.

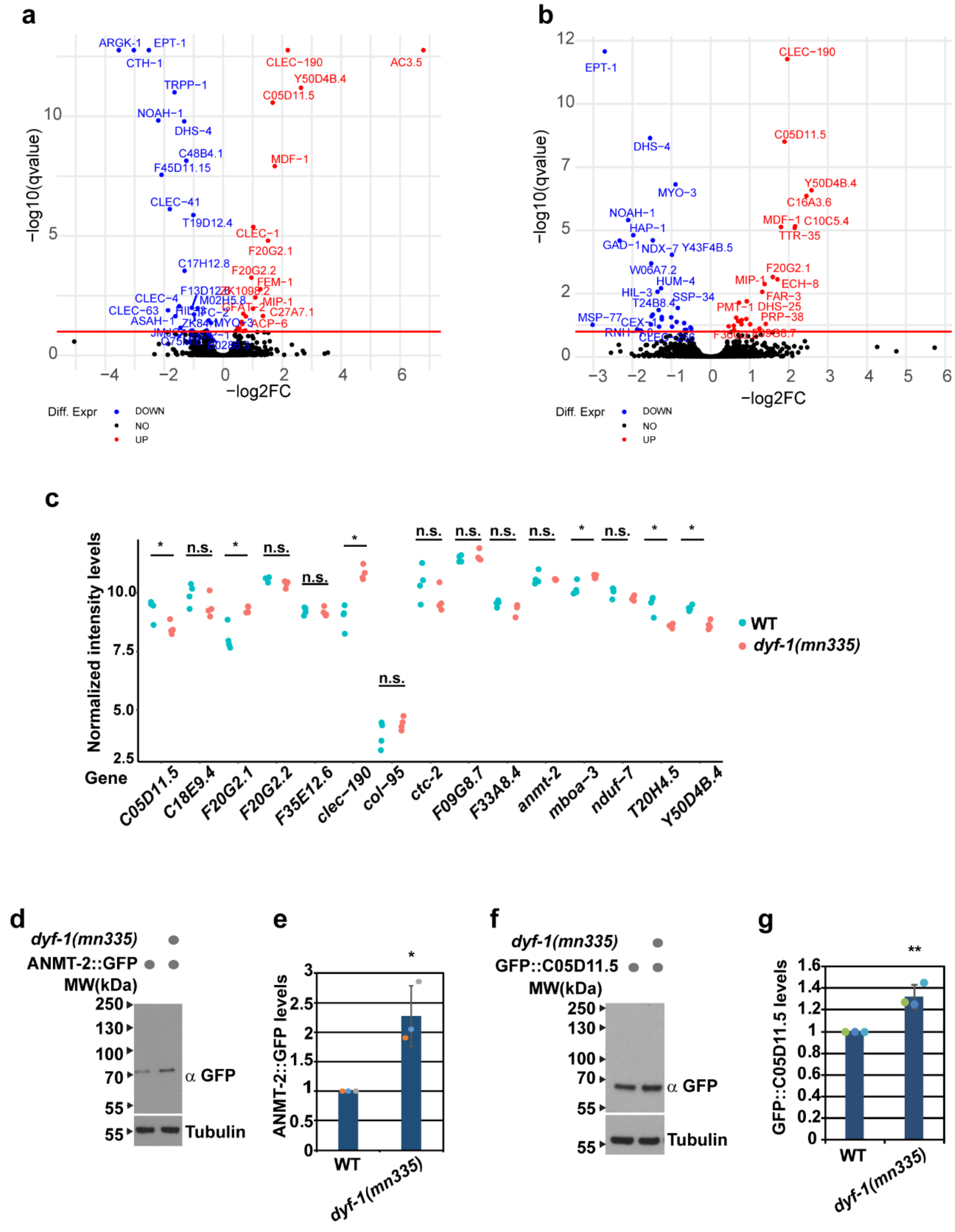

Supplementary Figure 3. Endogenous proteins affected by UPS dysfunction.

a Volcano plot showing significantly (q-value < 0.1) increased (red) or decreased (blue) proteins in the *dyf-1(mn335)* mutant compared with the *dyf-1(mn335); unc-31(e928)* mutant derived from

differential expression analysis of proteomic data. n = 3 biologically independent experiments. **b** Volcano plot of significantly (q-value < 0.1) increased (red) or decreased (blue) proteins in the *dyf-1(mn335)* mutant compared with the *dyf-1(mn335) unc-13(e51)* mutant, derived from differential expression analysis of proteomic data. n = 3 biologically independent experiments. **c** Gene expression levels of protein candidates regulated by neuronal signalling as displayed in Figure 3b derived by microarray analysis of the indicated strains. *C05D11.5* $p = 0.043$, *C18E9.6* $p = 0.083$, *F20G2.1* $p = 0.021$, *F20G2.2* $p = 0.083$, *F35E12.6* $p = 0.773$, *clec-190* $p = 0.021$, *col-95* $p = 0.248$, *ctc-2* $p = 0.149$, *F09G8.7* $p = 0.564$, *F33A8.4* $p = 0.149$, *anmt-2* $p = 0.772$, *mboa-3* $p = 0.021$, *nduf-7* $p = 0.149$, *T20H4.5* $p = 0.021$, *Y50D4B.4* $p = 0.021$, n. s. not significant, * $p \leq 0.05$, Kruskal Wallis test. **d** Worms expressing ANMT-2::GFP in the wild-type or *dyf-1(mn335)* mutant background were grown to day one of adulthood and protein lysates applied to Western blotting against GFP and tubulin, typical result from n = 3 biologically independent experiments. **e** Quantification of the experiments displayed in (d), n = 3 biologically independent experiments. $p = 0.01265$. **f** Worms expressing GFP::C05D11.5 were grown and analysed as in (d). **g** Quantification of the experiments displayed in (f), n = 3 biologically independent experiments, $p = 0.00688$. **e, g** 2-tailed unpaired Student's *t*- test. Column graphs: mean +/- standard deviation, scatter plots, individual experiments, same colour: same experiment, * $p \leq 0.05$, ** $p \leq 0.01$, n. s. $p > 0.05$. Source data are provided as a Source Data file.

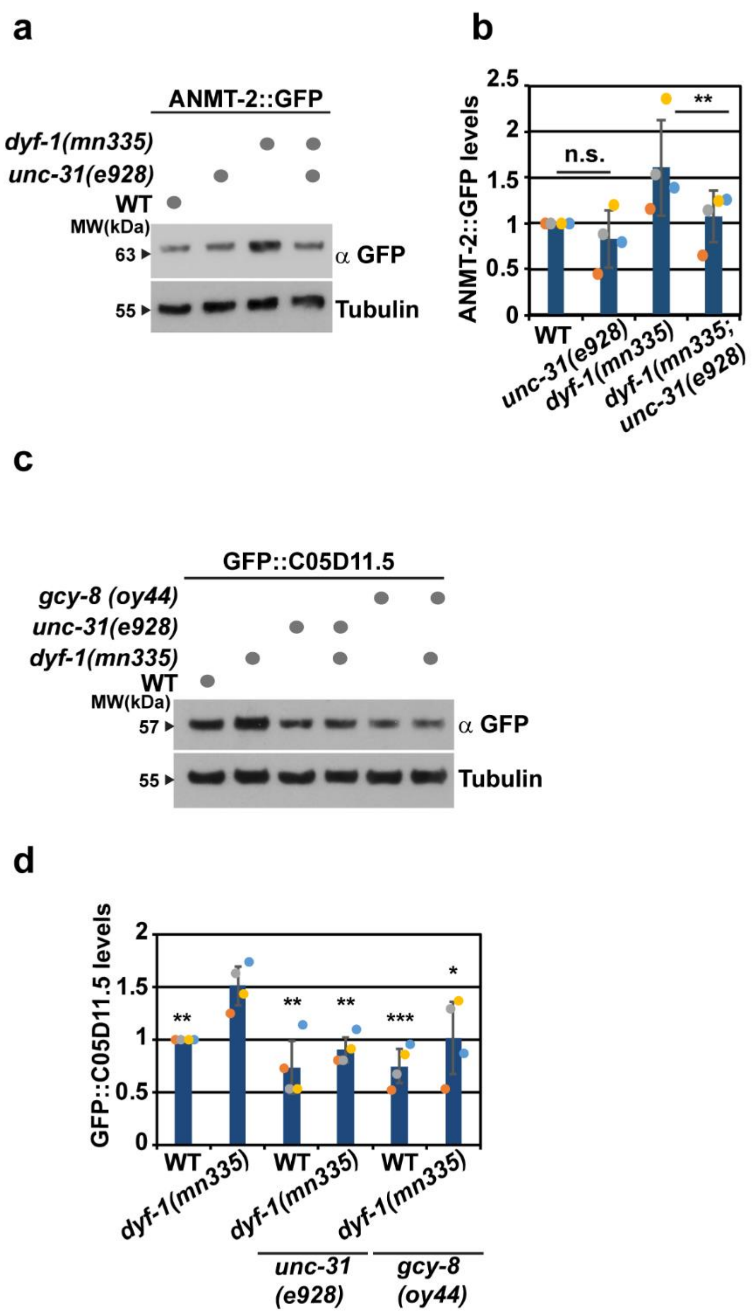

Supplementary Figure 4. Endogenous proteins affected by UPS dysfunction.

a Worms expressing ANMT-2::GFP in the wild-type or indicated mutant background were analysed by Western blotting against GFP and tubulin. **b** Quantification of the experiments displayed in (a), n = 4 biologically independent experiments, WT versus *unc-31(e928)* p = 0.15293, *dyf-1(mn335)* versus *dyf-1(mn335); unc-31(e928)* p = 0.005197, 1-tailed unpaired Student's t- test of indicated comparison when wild-type or *dyf-1(mn335)* is set to 1. Mean +/-

standard deviation, scatter plots: individual experiments, same colour: same experiment. **c** Worms expressing GFP::C05D11.5 in the wild-type or indicated mutant background analysed as in (a). **d** Quantification of the experiments displayed in (c), n = 4 biologically independent experiments, WT *p* = 0.00145, *unc-31(e928) p* = 0.00240, *dyf-1(mn335); unc-31(e928) p* =0.00144, *gcy-8(oy44) p* = 0.0009, *dyf-1(mn335); gcy-8(oy44) p* = 0.03401, 1-tailed unpaired Student's *t*- test compared with *dyf-1(mn335),* column graphs: mean +/- standard deviation, scatter plots: individual experiments, same colour: same experiment, *** p ≤ 0.001, ** p ≤ 0.01, * p ≤ 0.05, n.s. p > 0.05. Source data are provided as a Source Data file.

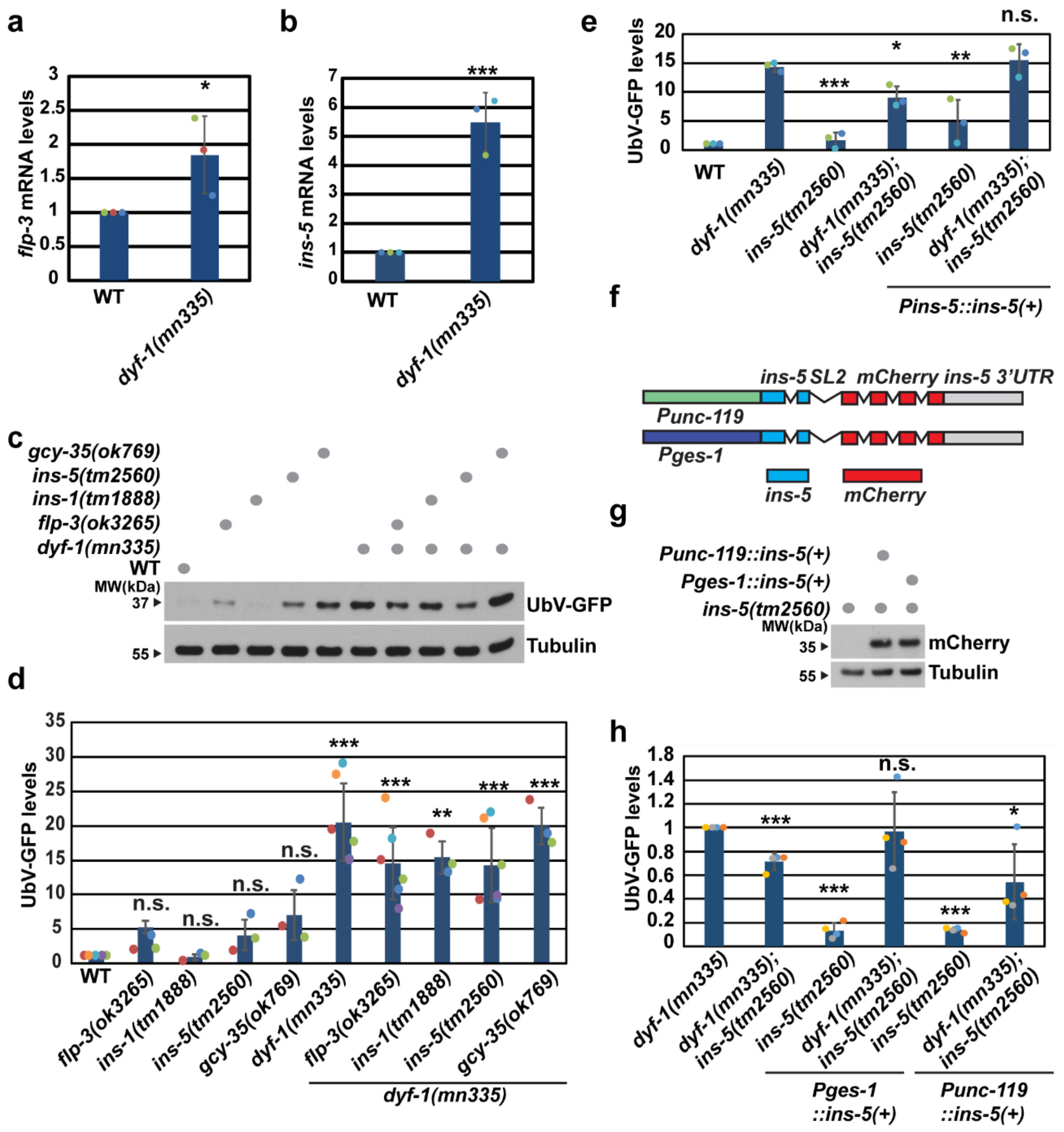

Supplementary Figure 5. INS-5 and FLP-3 control UFD degradation.

a Quantification of *flp-3* mRNA by qRT-PCR. p = 0.0309. **b** Quantification of *ins-5* mRNA by qRT-PCR. p = 0.00077, (a, b) n = 3 biologically independent experiments, 1-tailed unpaired Student's *t*- test. Column graphs: mean +/- standard deviation, scatter plots: individual experiments. **c** Indicated mutant worms expressing the UFD substrate were analysed as in Figure 4c. **d** Quantification of worms displayed in (c). Column graphs: mean +/- standard deviation, scatter plots: individual experiments, 6 (wild-type, *dyf-1(mn335), dyf-1(mn335); flp-3(ok3265), dyf-1(mn335); ins-5(tm2560)*) all other 3 biologically independent experiments, *flp-3(ok3265)* p =

0.06136, *ins-1(tm1888) p* = 0.79612, *ins-5(tm2560) p* = 0.11915, *gcy-35(ok769) p* = 0.08019, *dyf-1(mn335) p* < 0.0001, *dyf-1(mn335); flp-3(ok3265) p* = 0.00040, *dyf-1(mn335); ins-1(tm1888)* p = 0.00107, *dyf-1(mn335); ins-5(tm2560) p* = 0.00019, *dyf-1(mn335); gcy-35(ok769) p* = 0.0005, 2-tailed unpaired Student's *t*- test versus wild-type. **e** Quantification of UFD substrate levels of indicated mutant worms, n = 3 biologically independent experiments, *ins-5(tm2560) p* = 0.0003, *dyf-1(mn335); ins-5(tm2560) p* = 0.0283, *ins-5(tm2560); Pins-5::ins-5(+) p* = 0.0027, *dyf-1(mn335); ins-5(tm2560); Pins-5::ins-5(+) p* = 0.6873, one-way ANOVA followed by Dunnett's multiple comparisons test compared with *dyf-1(mn335)* which was set to one. Column graphs: mean +/- standard deviation, scatter plots: individual experiments, same colour: same experiment. **f** Schematic overview of tissue-specific *ins-5* rescue constructs. **g** Western blot of the *ins-5 (tm2560)* mutant expressing the tissue-specific rescue constructs. **h** Quantification of UFD levels of the indicated mutant worms. n = 4 biologically independent experiments, *dyf-1(mn335); ins-5(tm2560) p* = 0.00019, *ins-5 (tm2560); Pges-1::ins-5(+) p* < 0.0001, *dyf-1(mn335); ins-5 (tm2560); Pges-1::ins-5(+) p* = 0.85336, *ins-5 (tm2560); Punc-119::ins-5(+) p* < 0.0001, *dyf-1(mn335); ins-5 (tm2560); Punc-119::ins-5(+) p* = 0.02682, 2-tailed unpaired Student's *t*- test compared with *dyf-1(mn335).* Column graphs: mean +/- standard deviation, scatter plots: individual experiments, same colour: same experiment *** p ≤ 0.001, ** p ≤ 0.01, * p ≤ 0.05, n.s. p > 0.05. Source data are provided as a Source Data file.

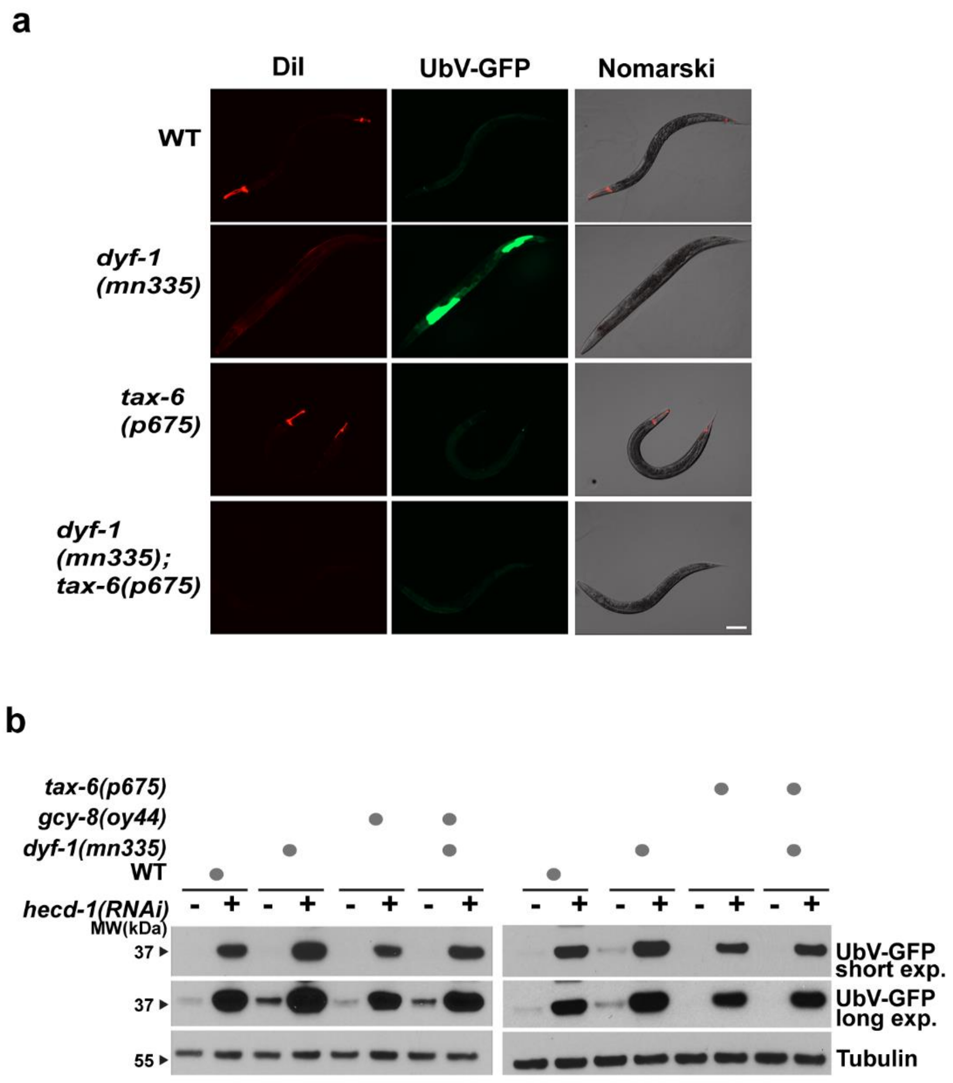

Supplementary Figure 6. TAX-6 and GCY-8 affect UFD degradation dependent of HECD-1. **a** Fluorescence and Nomarski images of worms expressing the UFD substrate in wild-type, *dyf-1(mn335), tax-6(p675)* or *dyf-1(mn335); tax-6(p675)* mutant worms. DiI denotes ciliated amphid and phasmid neurons. n = 2 biologically independent experiments. Scale bar: 100 μm. **b** Protein lysates of worms were analysed by SDS-PAGE and immunoblotting against GFP and tubulin as loading control. Dots denote presence of indicated mutations, worms were grown on control (-) or *hecd-1(RNAi)* (+) plates, n = 3 (left panel) or 2 (right panel) biologically independent experiments. Source data are provided as a Source Data file.

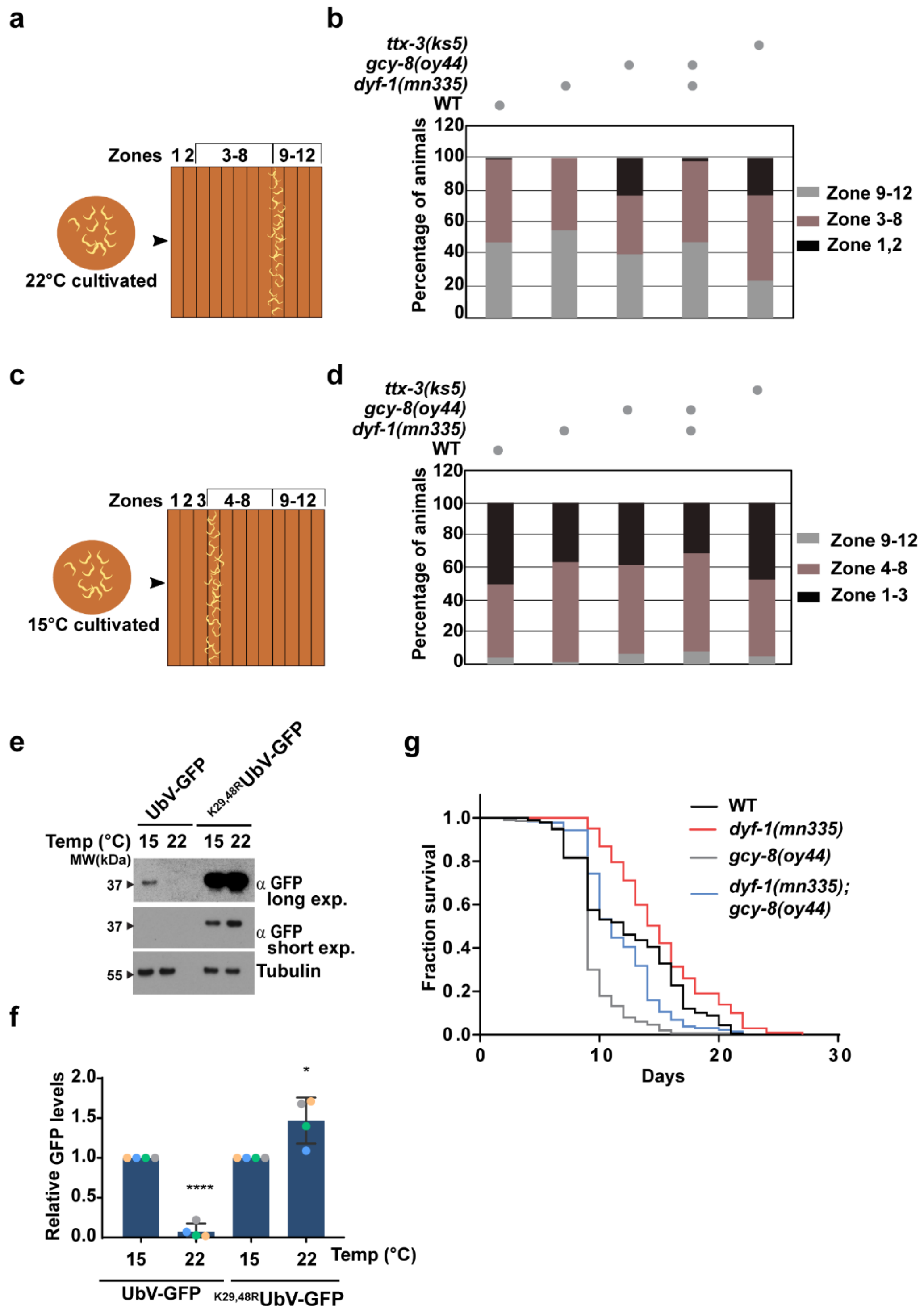

Supplementary Figure 7. Temperature-independent behaviour of the sensory mutants.

a Movement of animals in the absence of a linear thermal gradient was measured on a 12*12 cm plate at room temperature. The plate was divided into 12 regions (zone 1-12). Animals were

cultivated at 22°C followed by placement in the region named zone 9 to imitate the position of the starting area used in Figure 6b, c (Tstart). **b** Number of indicated mutant animals analysed are wild-type 248, *dyf-1(mn335)* 162, *gcy-8(oy44)* 128, *dyf-1(mn335); gcy-8(oy44)* 192 and *ttx-3(ks5)* 129, n = 1. **c, d** similar as (a, b) but animals were cultivated at 15°C followed by placement in the region named as Zone 4 to imitate the position of the starting area (Tstart) used in Figure 6d, e. Number of animals are wild-type 249, *dyf-1(mn335)* 169, *gcy-8(oy44)* 198, *dyf-1(mn335); gcy-8(oy44)* 201 and *ttx-3(ks5)* 179. The percentage distribution of animals in the specified zones are indicated, n = 1. **e** Protein lysates of worms expressing UbV-GFP or the nondegradable ^{K29,48R}UbV-GFP grown at 15°C or 22°C and analysed by SDS-PAGE and immunoblotting against GFP and tubulin as loading control. long exp., short exp.: long and short exposure of the blot, respectively. **f** Quantification of UFD levels of the experiment shown in (e). n = 4 biologically independent experiments, 2-tailed unpaired Student's *t*- test of strains expressing either UbV-GFP ($p < 0.0001$) or ^{K29,48R}UbV-GFP ($p = 0.0175$) in WT at 15 °C compared with WT at 22°C**.** Column graphs: Mean +/- standard deviation, scatter plots: individual experiments. **** p ≤ 0.0001, * p ≤ 0.05. **g** Lifespan analysis of worms as indicated grown at 22°C on NGM containing OP50. n = 4 biologically independent experiments. Detailed statistics including *p* values can be found in Supplementary Table 5. Source data are provided as a Source Data file.

Supplementary Table 1. UbV-GFP stability after RNAi treatment in the neuron-sensitive RNAi mutant background.

GENE	UbV-GFP signal in *dyf-1(mn335); nrh-1(hd20)lin-15b(hd126)*
acr-19	+
acr-23	+
acr-5	+
flp-16	+
flp-21	+
gcy-18	-
gcy-22	+
gcy-33	+
gcy-35	+ -
glr-1	+
glr-5	++
gpa-4	+
gpa-6	+
gpa-7	+ -
ida-1	+ -
ins-1	+ -
ins-5	-
nlp-13	+
nlp-14	+
nlp-37	+
nlp-8	+
odr-3	+
ser-4	+
srr-1	++
srd-32	+
srd-60	++
syd-9	+
syg-1	+
syg-2	+
tax-2	+
tax-6	-
unc-2	+
unc-30	+
unc-43	++
unc-47	+
unc-49	+
unc-7	+
unc-9	+
dop-5	++
ceh-36	+
flp-3	+ -
flp-2	+
gbb-2	+
glr-3	+
mgl-2	+
nlp-18	+
nlp-9	+
odr-2	+
ser-2	+
ser-1	+
mod-1	+
syd-1	+
egl-3	+
kpc-1	+
egl-8	+
egl-30	++
dop-1	++
cat-1	+
dgk-3	+
rab-3	+
rab-8	+
acy-1	+
ncx-1	+
gsa-1	+

Supplementary Table 2. Thermotaxis statistics Tc 22°C. Analysis of categorical data using a one-tailed probability of the Chi-squared distribution with an alpha value of $p < 0.05$.

Counts of animals in each category from three independent experiments are denoted below for Figure 6c. A chi-square test is used to investigate whether the distribution of animals by mutations differed in these three temperature categories. The null hypothesis states that there is no difference between the two distributions.			
Strain	24°C onwards	18.5°C - 23.5°C	< 18.5°C
WT	42	340	170
dyf-1(mn335)	680	413	24
gcy-8(oy44)	202	365	192
dyf-1(mn335); gcy-8(oy44)	340	431	39
ttx-3(ks5)	25	90	700
Chi-square statistics for all 5 strains			
Level of significance		0.05	
Number of rows		5	
Number of columns		3	
Degrees of freedom		8	
Critical value		15.507	
Chi-square statistic		2338.325	
p-value		0, <0.00001	
Chi-square statistics for WT and *dyf-1(mn335)*			
Level of significance		0.05	
Number of rows		2	
Number of columns		3	
Degrees of freedom		2	
Critical value		5.991	
Chi-square statistic		552.811	
p-value		9.09e^-121, <0.00001	
Chi-square statistics for *dyf-1(mn335)* and *dyf-1(mn335); gcy-8(oy44)*			
Level of significance		0.05	
Number of rows		2	
Number of columns		3	
Degrees of freedom		2	
Critical value		5.991	
Chi-square statistic		70.159	
p-value		5.82e^-16, <0.00001	

Supplementary Table 3. Thermotaxis statistics Tc 15°C. Analysis of categorical data using a one-tailed probability of the Chi-squared distribution with an alpha value of $p < 0.05$

Counts of animals in each category from three independent experiments are denoted below for Figure 6e. As the counts in category 24°C onwards is < 5 in some rows, this category was excluded. The chi-square test was used to investigate whether the distribution of wild-type and mutant animals differed in two temperature categories namely 18°C - 23.5°C and 17.5°C onwards. The null hypothesis states that there is no difference between distributions.			
Strain	24°C onwards	18°C - 23.5°C	17.5°C onwards
WT	1	102	466
dyf-1(mn335)	5	356	330
gcy-8(oy44)	7	119	371
dyf-1(mn335); gcy-8(oy44)	6	190	314
ttx-3(-)	0	60	521
Chi-square statistics for all 5 strains			
Level of significance		0.05	
Number of rows		5	
Number of columns		2	
Degrees of freedom		4	
Critical value		9.487	
Chi-square statistic		328.849	
p-value		6.45507e^-70, <0.00001	
Chi-square statistics for WT and *dyf-1(mn335)*			
Level of significance		0.05	
Number of rows		2	
Number of columns		2	
Degrees of freedom		1	
Critical value		3.841	
Chi-square statistic		154.363	
p-value		1.92836e^-35, <0.00001	
Chi-square statistics for *dyf-1(mn335)* and *dyf-1(mn335); gcy-8(oy44)*			
Level of significance		0.05	
Number of rows		2	
Number of columns		2	
Degrees of freedom		1	
Critical value		3.841	
Chi-square statistic		23.582	
p-value		1.19658e^-06, <0.00001	

Supplementary Table 4. Thermotaxis statistics Tc 22°C. Analysis of categorical data using a one-tailed probability of the Chi-squared distribution with an alpha value of $p < 0.05$

Counts of animals in each category from three independent experiments are denoted below. A chi-square test is used to investigate whether the distribution of animals by mutations (Fig. 6f) differed in these three temperature categories. The null hypothesis states that there is no difference between the two distributions.			
Strain	24°C onwards	18.5°C - 23.5°C	< 18.5°C
WT	49	547	58
dyf-1(mn335)	243	579	9
str-2(tm445) odr-7(tm4791)	205	811	193
gcy-8(oy44); str-2(tm445) odr-7(tm4791)	31	404	285
ttx-3(ks5)	25	236	717
Chi-square statistics for all 5 strains			
Level of significance		0.05	
Number of rows		5	
Number of columns		3	
Degrees of freedom		8	
Critical value		15.507	
Chi-square statistic		1718.6	
p-value		0, <0.000001	
Chi-square statistics for WT and *dyf-1(mn335)*			
Level of significance		0.05	
Number of rows		2	
Number of columns		3	
Degrees of freedom		2	
Critical value		5.991	
Chi-square statistic		146.6	
p-value		1.45 e^-32, <0.00001	
Chi-square statistics for WT and *str-2(tm445); odr-7(tm4791)*			
Level of significance		0.05	
Number of rows		2	
Number of columns		3	
Degrees of freedom		2	
Critical value		5.991	
Chi-square statistic		59.7	
p-value		1.085e^-13, <0.00001	
Chi-square statistics for *str-2(tm445); odr-7(tm4791)* and *str-2(tm445); odr-7(tm4791); gcy-8(oy44)*			
Level of significance		0.05	
Number of rows		2	
Number of columns		3	
Degrees of freedom		2	
Critical value		5.991	
Chi-square statistic		169.3	
p-value		1.77e^-37, <0.00001	

Supplementary Table 5. Statistical analysis of lifespan at 22°C.

Lifespan Statistics for all 4 experiments combined						
**Strain name**	**Mean**	**sd**	**n**	**SEM**	**p-value (t-Test, two-sided, unpaired)**	**p-value (Mantel cox log-rank test, two-sided)**
WT	12.21	4.603	164	0.3594		
gcy-8(oy44)	9.248	2.32	165	0.1806	WT <0.0001<br>*dyf-1(mn335); gcy-8(oy44)* <0.0001	WT <0.0001<br>*dyf-1(mn335); gcy-8(oy44)* <0.0001
dyf-1(mn335);gcy-8(oy44)	11.51	3.23	144	0.2692	WT 0.1320	WT 0.0454
dyf-1(mn335)	14.76	4.029	114	0.3774	WT <0.0001<br>*dyf-1(mn335); gcy-8(oy44)* <0.0001	WT <0.0001<br>*dyf-1(mn335); gcy-8(oy44)* <0.0001
**Experiment 1**						
WT	12.32	4.598	38	0.7459		
gcy-8(oy44)	8.821	2.416	39	0.3868	WT <0.0001<br>*dyf-1(mn335); gcy-8(oy44)* <0.0001	WT <0.0001<br>*dyf-1(mn335); gcy-8(oy44)* <0.0001
dyf-1(mn335);gcy-8(oy44)	11.74	3.592	38	0.5827	WT 0.5426	WT 0.3751
dyf-1(mn335)	13.92	2.799	26	0.5489	WT 0.1169<br>*dyf-1(mn335); gcy-8(oy44)* 0.0114	WT < 0.4370<br>*dyf-1(mn335); gcy-8(oy44)* 0.0068
**Experiment 2**						
WT	11.9	4.963	40	0.7848		
gcy-8(oy44)	8.814	1.577	43	0.2406	WT 0.0002<br>*dyf-1(mn335); gcy-8(oy44)*<0.0001	WT 0.0006<br>*dyf-1(mn335); gcy-8(oy44)* <0.0001
dyf-1(mn335);gcy-8(oy44)	11.86	3.025	36	0.5042	WT<br>0.9469	WT<br>0.4897
dyf-1(mn335)	17.75	4.926	28	0.931	WT <0.0001<br>*dyf-1(mn335); gcy-8(oy44)* <0.0001	WT <0.0001<br>*dyf-1(mn335); gcy-8(oy44)* <0.0001
**Experiment 3**						
WT	13.1	4.601	44	0.6937		
gcy-8(oy44)	9.595	3.037	42	0.4686	WT <0.0001<br>*dyf-1(mn335); gcy-8(oy44)* 0.0449	WT <0.0001<br>*dyf-1(mn335); gcy-8(oy44)* 0.0052
dyf-1(mn335);gcy-8(oy44)	11.15	3.594	34	0.6164	WT 0.0435	WT 0.1022
dyf-1(mn335)	13.73	3.233	33	0.5629	WT 0.5151<br>*dyf-1(mn335); gcy-8(oy44)* 0.0030	WT < 0.9816<br>*dyf-1(mn335); gcy-8(oy44)* 0.0185
**Experiment 4**						
WT	11.43	4.232	42	0.653		
gcy-8(oy44)	9.756	1.921	41	0.3	WT 0.0235<br>*dyf-1(mn335); gcy-8(oy44)* 0.0053	WT 0.0102<br>*dyf-1(mn335); gcy-8(oy44)* 0.0047
dyf-1(mn335);gcy-8(oy44)	11.28	2.7	36	0.4501	WT 0.8545	WT 0.3994
dyf-1(mn335)	13.74	3.493	27	0.6722	WT 0.0209<br>*dyf-1(mn335); gcy-8(oy44)* 0.0025	WT 0.0309<br>*dyf-1(mn335); gcy-8(oy44)* 0.0020

Supplementary Table 6. Key reagents and resources.

REAGENT or RESOURCE	SOURCE	IDENTIFIER
**Antibodies**		
Mouse monoclonal anti alpha tubulin (clone B-5-1-2)	Sigma	Cat#T6074; RRID:AB_477582
Living Colors, A.v. Monoclonal Antibody (JL-8) , anti-GFP	Clontech Laboratories, Inc.	Cat# 632380; RRID:AB_10013427
anti mCherry, mouse Monoclonal Antibody (1C51), IgG2a	Abcam	Cat# ab125096; RRID:AB_11133266
Peroxidase-conjugated AffiniPure Goat Anti- Mouse IgG + IgM (H+L)	Jackson ImmunoResearch	Cat# 211-035-109; RRID: AB_2339150
Donkey anti-mouse IRDye® 800CW/680	LI-COR	Cat# 926-32212 RRID:AB_621847
**Bacterial strains**		
E. coli OP50	CGC	RRID:WB-STRAIN:WBStrain00041969
E. coli C600	Hyman A, MPI of Molecular Cell Biology & Genetics, Dresden, GER	N/A
E. coli HT115(DE3)	CGC	N/A
E.coli DH5α™	Invitrogen	Cat# 18265017
NEB 5-alpha Competent *E. coli* (High Efficiency)	New England Biolabs	Cat# C2987H
Bacterial *C. elegans* RNAi Collection (Ahringer)	Source BioScience Ltd	RRID:SCR_017064
Bacterial *C. elegans* RNAi Collection (ORFeomeWS112)	Geneservice Ltd, available via Source BioScience	Laboratory of Marc Vidal
**Critical commercial assays**		
High-Capacity cDNA Reverse Transcription Kit	Applied Biosystems	Cat# 4368814
RNeasy® Mini Kit	Qiagen	Cat# 74104
NEBuilder® HiFi DNA Assembly Cloning Kit	NEB	Cat# E5520S
**Deposited Data**		
Microarray data	This paper	GSE142371
Proteomics data	This paper	PXD016676
**Recombinant DNA**		
pTH1219 pBS-*unc-119-Pins-5-ins-5-SL2-NLS-mCherry-ins5 3'UTR*	This paper	N/A
pTH1707 pBS-*unc-119-Pges-1-ins-5-SL2-NLS-mCherry-ins-5 3'UTR*	This paper	N/A
pTH1720 pBS-unc-119-*Punc-119-ins-5-SL2-NLS-mCherry-ins-5 3'UTR*	This paper	N/A
pBalu12	[1]	N/A
pCC1FOS-anmt-2	Source Bioscience	Prod# CBGtg9050F039D
**Software and algorithms**		
GraphPad Prism 7	GraphPad Software, Inc.	https://www.graphpad.com/scientific-software/prism/
Image J 1.48v	Wayne Rasband (NIH)	https://imagej.nih.gov/ij/
Excel 2016	Microsoft	https://products.office.com/en-us/excel
Image Studio 4.0	LI-COR Biosciences	https://www.licor.com/bio/products/software/image_studio/
Zen 2.3 pro	Zeiss	https://www.zeiss.com/microscopy/us/products/microscope-software/zen.html
ZEN connect modul	Zeiss	https://www.zeiss.com/microscopy/int/products/microscope-software/zen-connect-image-overlay-and-correlative-microscopy.html?vaURL=www.zeiss.com/zen-connect
Leica application Suite 3.3.1	Leica	https://www.leica-microsystems.com/products/microscope-software/p/leica-application-suite/
Adobe Illustrator v26.5	Adobe	https://www.adobe.com/de/#
KEGG	[2]	https://www.genome.jp/kegg/
Adobe Photoshop v23.4.2	Adobe	https://www.adobe.com/products/photoshop.html?promoid=PC1PQQ5T&mv=other
Inkscape vector graphics editor 1.1	Open Source	https://inkscape.org/
SnapGene® 4.0.8.0	GSL Biotech LLC	http://www.snapgene.com/
MaxQuant 1.5.3.8	[3]	https://www.maxquant.org/
Bio-Rad CFX Manager™	Bio-Rad Laboratories Inc.	http://www.bio-rad.com/de-de/sku/1845000-cfx-manager-software
ReactomePA 1.34.0	Bioconductor	https://bioconductor.org/packages/release/bioc/html/ReactomePA.html
R v3.4.3	[4]	https://www.r-project.org/
oligo v1.42.0	Bioconductor	https://www.bioconductor.org/packages/release/bioc/html/oligo.html
limma v3.34.9	Bioconductor	https://bioconductor.org/packages/release/bioc/html/limma.html
DEP v1.8.0	Bioconductor	https://bioconductor.org/packages/release/bioc/html/DEP.html
pathview v1.18.2	Bioconductor	https://bioconductor.org/packages/release/bioc/html/pathview.html

Supplementary References

1 Tursun, B., Cochella, L., Carrera, I. & Hobert, O. A toolkit and robust pipeline for the generation of fosmid-based reporter genes in C. elegans. *PLoS One* **4**, e4625, doi:10.1371/journal.pone.0004625 (2009).

2 Kanehisa, M. A database for post-genome analysis. *Trends Genet* **13**, 375-376, doi:10.1016/s0168-9525(97)01223-7 (1997).

3 Cox, J. & Mann, M. MaxQuant enables high peptide identification rates, individualized p.p.b.-range mass accuracies and proteome-wide protein quantification. *Nat Biotechnol* **26**, 1367-1372, doi:10.1038/nbt.1511 (2008).

4 R: A language and environment for statistical computing. (R Foundation for Statistical Computing, Vienna, Austria, 2017).
