# Supplementary material for: Thermosensation in Caenorhabditis elegans is linked to ubiquitin-dependent protein turnover via insulin and calcineurin signalling
Source: Nat Commun. 2022 Oct 5;13:5874. doi: 10.1038/s41467-022-33467-7 (PMC9534930; doi:10.1038/s41467-022-33467-7)
Supplement: Supplementary file 3 — Description of Additional Supplementary Files [file 41467_2022_33467_MOESM3_ESM.docx]

**Description of Additional Supplementary Files**

**Supplementary Data 1:** Proteomics analysis

**Supplementary Data 2:** Microarray analysis

**Supplementary Data 3:** *C. elegans* strains

**Supplementary Data 4:** Oligos used in this study
