# Supplementary material for: Thermosensation in Caenorhabditis elegans is linked to ubiquitin-dependent protein turnover via insulin and calcineurin signalling
Source: Nat Commun. 2022 Oct 5;13:5874. doi: 10.1038/s41467-022-33467-7 (PMC9534930; doi:10.1038/s41467-022-33467-7)
Supplement: Supplementary file 8 — Reporting Summary [file 41467_2022_33467_MOESM8_ESM.pdf]

nature portfolio

Corresponding author(s): Thorsten Hoppe
Alexandra Segref

Last updated by author(s): Aug 22, 2022

Reporting Summary

Nature Portfolio wishes to improve the reproducibility of the work that we publish. This form provides structure for consistency and transparency in reporting. For further information on Nature Portfolio policies, see our Editorial Policies and the Editorial Policy Checklist.

Statistics

For all statistical analyses, confirm that the following items are present in the figure legend, table legend, main text, or Methods section.

n/a	Confirmed	
☐	☒	The exact sample size (n) for each experimental group/condition, given as a discrete number and unit of measurement
☐	☒	A statement on whether measurements were taken from distinct samples or whether the same sample was measured repeatedly
☐	☒	The statistical test(s) used AND whether they are one- or two-sided<br>*Only common tests should be described solely by name; describe more complex techniques in the Methods section.*
☒	☐	A description of all covariates tested
☐	☒	A description of any assumptions or corrections, such as tests of normality and adjustment for multiple comparisons
☐	☒	A full description of the statistical parameters including central tendency (e.g. means) or other basic estimates (e.g. regression coefficient) AND variation (e.g. standard deviation) or associated estimates of uncertainty (e.g. confidence intervals)
☐	☒	For null hypothesis testing, the test statistic (e.g. F, t, r) with confidence intervals, effect sizes, degrees of freedom and P value noted<br>*Give P values as exact values whenever suitable.*
☒	☐	For Bayesian analysis, information on the choice of priors and Markov chain Monte Carlo settings
☒	☐	For hierarchical and complex designs, identification of the appropriate level for tests and full reporting of outcomes
☒	☐	Estimates of effect sizes (e.g. Cohen's d, Pearson's r), indicating how they were calculated

Our web collection on statistics for biologists contains articles on many of the points above.

Software and code

Policy information about availability of computer code

Data collection	Image Studio 4.0 for some Western blots, Zen 2.3 pro , ZEN connect modul, Leica application Suite 3.3.1 for images, Bio-RadCFX Manager 3.0 for qRT-PCR.
Data analysis	Image J 1.48v and Image Studio 4.0, Graph pad 5 and 7, Microsoft Excel 2016 for quantifications, R v3.4.3, limma v3.34.9 R, ReactomePA 1.34.0 ,KEGG, pathview v1.18.2 for microarray analysis, Max quant version 1.5.3.8 and DEP package v1.8.0 for Mass spec analysis. Inkscape vector graphics editor 1.1, Adobe Illustrator v26.5, Adobe Photoshop v23.4.2, SnapGene® 4.0.8.0 for DNA analysis, Code: https://github.com/nevelsk90/Celegans_sensingDyf1.git . Detailed information is provided in the Methods.

For manuscripts utilizing custom algorithms or software that are central to the research but not yet described in published literature, software must be made available to editors and reviewers. We strongly encourage code deposition in a community repository (e.g. GitHub). See the Nature Portfolio guidelines for submitting code & software for further information.

Data

Policy information about availability of data

All manuscripts must include a data availability statement. This statement should provide the following information, where applicable:
- Accession codes, unique identifiers, or web links for publicly available datasets
- A description of any restrictions on data availability
- For clinical datasets or third party data, please ensure that the statement adheres to our policy

Source data are provided with this paper. All data needed to evaluate the conclusion are available in the main text or the supplementary data files provided with this manuscript. Mutant strains are available upon request from the authors and/or CGC. The microarray data generated in this study have been deposited under the accession code GSE142371 in the Gene expression omnibus database (GEO, NCBI) and are publicly available: https://www.ncbi.nlm.nih.gov/geo/query/acc.cgi?

acc=GSE142371. The Gene ontology database is available via: http://geneontology.org/, the Reactome database is available via: https://bioconductor.org/packages/release/bioc/html/ReactomePA.html. The Uniprot reference C. elegans proteome database is available at: https://www.uniprot.org/proteomes/UP000001940. The proteomics data have been deposited with the identifier PXD016676 via the ProteomeXchange database and are publicly available: https://www.ebi.ac.uk/pride/archive/projects/PXD016676.

Field-specific reporting

Please select the one below that is the best fit for your research. If you are not sure, read the appropriate sections before making your selection.

- [x] Life sciences
- [ ] Behavioural & social sciences
- [ ] Ecological, evolutionary & environmental sciences

For a reference copy of the document with all sections, see nature.com/documents/nr-reporting-summary-flat.pdf

Life sciences study design

All studies must disclose on these points even when the disclosure is negative.

Sample size	No statistical methods were used to predetermine sample size. Exact sample sizes are stated in the Figure legends, Materials and Methods, and Supplementary Information. For Western blot analysis 100 individuals per condition were used as established previously (Segref et al. Genetics 187, 1235-1240, 2011, Methods Mol Biol 832, 531-544, 2012 and Cell Metab. Apr 1;19(4):642-52, 2014, Finger et al Nat Metab 1, 350-359. 2019). For thermotaxis, 300 worms were used similar to Goodman et al. Wormbook. 2014doi/10.1895/wormbook.1.168.1. For microarrya data 200 - 300 worms were collected as published previously (Finger et al Nat Metab 1, 350-359. 2019)), for Lifespan 50 worms were evaluated per experiment as commonly described in the C elegans field and published previously(Cornwell, A.B., Samuelson, A.V. (2020), Methods in Molecular Biology, vol 2144. https://doi.org/10.1007/978-1-0716-0592-9_2, Kuhlbrodt et al. Nat. Cell Biol. 13, 273-81.2011, Tawo et al.Cell 169, 470-82.2017). For proteomic analysis sample size was determined reducing biological variability while ensuring similar protein lysate amounts and to ensure maximal loading of the stage tips for peptide separation according to previous C. elegans proteomics work (Lee H.J. et al, Nature Metabolism 1, 790-810 (2019). For microscopy sample size was used similar the previous publication Prahlad, V. & Morimoto, R. I. Proc Natl Acad Sci U S A 108, 14204-14209.2011, Maurya, A.K. Current Biology, Volume 29, Issue 8, 22 April 2019, Pages 1286-1300.e4).
Data exclusions	For lifespan analysis worms that crawled up the plate walls and desiccated or exhibited a 'protruding vulva' or showed 'bagging' were excluded from the study. For thermotaxis experiment worms which crawled up the plate walls and dried up during the 60 minutes of the assay were censored from the study.
Replication	Multiple biological replicates of each experiment were conducted as indicated in the figure legends.
Randomization	Worms grouped per genotype were bleached by the standard method and placed randomly on growth plates. To synchronise worms, L3 worms were randomly picked under the stereomicroscope onto new plates. Day 1 adult worms were then randomly collected and analysed either by Westernblotting, collection of RNA, by fluorescence microscopy, thermotaxis assay or lifespan.
Blinding	For Western blotting, qPCR no blinding was performed since the analysis of the results are obtained by objective measurements. Microscopy experiments were not blinded, but measurements and phenotypes examined by multiple investigators to confirm the observed phenotypes. The lifespan experiment was blinded during data collection. The thermotaxis experiment was performed blinded during data collection at 22 °C to set up the assay. During later experiments the ttx-3 mutant served as control to ensure that the gradient machine setup worked, therefore the experiments were not blinded.<br>For proteomics and microarray analysis the experiments were not blinded since the mutant phenotype (UFD stabilisation) was observed under a microscope to ensure the correct experimental setup. Further processing of the samples was performed by independent researchers in the proteomics and genomics core facilities in a blinded manner. Blinded analysis was not sensible as it required an integrative evaluation of the obtained data.

Reporting for specific materials, systems and methods

We require information from authors about some types of materials, experimental systems and methods used in many studies. Here, indicate whether each material, system or method listed is relevant to your study. If you are not sure if a list item applies to your research, read the appropriate section before selecting a response.

Materials & experimental systems

n/a	Involved in the study	
	x	Antibodies
x		Eukaryotic cell lines
x		Palaeontology and archaeology
	x	Animals and other organisms
x		Human research participants
x		Clinical data
x		Dual use research of concern

Methods

n/a	Involved in the study	
x		ChIP-seq
x		Flow cytometry
x		MRI-based neuroimaging

Antibodies

Antibodies used	Mouse monoclonal anti alpha tubulin (clone B-5-1-2) Sigma-Aldrich,Cat#T6074 ; RRID:AB_477582, 1:5000, Living Colors, A.v. Monoclonal Antibody (JL-8) , anti-GFP ,Clontech Laboratories, Cat# 632380; RRID:AB_10013427, 1:5000,<br>anti mCherry, mouse Monoclonal Antibody (1C51), IgG2a, Abcam,Cat# ab125096; RRID:AB_11133266, 1:2000<br>Peroxidase-conjugated AffiniPure Goat Anti- Mouse IgG + IgM, Jackson Immunoresearch, Cat# 211-035-109; RRID: AB_2339150, 1:10000<br>Donkey anti-mouse IRDye® 800CW/680, LI-COR, Cat# 926-32212 RRID:AB_621847, 1:10 000
Validation	All validations for the primary antibodies were performed by the manufacturer and can be found on the manufacturer's websites and via the Research Resource Identifiers.<br>Anti-tubulin, Sigma-Aldrich,Cat#T6074 ; RRID:AB_477582:<br>Company description:<br>around 50 kDa reacts with alpha tubulin, alpha bovine, chicken/avian, human, mouse, other, rat, simian, human, mouse, bovine, rat, african green monkey, kangaroo rat, chicken, sea urchin, chlamydomonas, amoeba/protozoa, other invertebrate<br>We confirmed the correct size band by Western blotting and used it as a loading control for western blotting in this manuscript.<br><br>Anti-GFP, Clontech Laboratories, Cat# 632380; RRID:AB_10013427:<br>Company description<br>A monoclonal antibody produced by hybridoma cells against full-length Aequorea victoria green fluorescent protein (GFP). This antibody recognizes native and denatured forms of wild-type GFP, GFPuv, AcGFP, EGFP, destabilized EGFP variants, EBFP, EYFP, ECFP, AcGFP, and both N- and C-terminal fusion proteins containing these GFP variants in bacterial and mammalian cell lysates.<br>We have previously confirmed that this antibody detects the correct size bands of GFP or Ub-GFP by western blotting of C. elegans extracts that express GFP or Ub-GFP as compared to wild-type C. elegans (Segref et al. Genetics 187, 1235-1240, 2011, Methods Mol Biol 832, 531-544, 2012 and Cell Metab. Apr 1;19(4):642-52, 2014).<br><br>Anti mCherry, Abcam,Cat# ab125096; RRID:AB_11133266, .<br>Company description: Mouse monoclonal [1C51] to mCherry, Reacts with: species independent.<br>We confirmed the detection of the correct size band in worms expressing mCherry by western blotting as compared to N2 worms not expressing mCherry previously (Segref et al. Genetics 187, 1235-1240, 2011) and in this manuscript (Supplementary Fig. 5g).

Animals and other organisms

Policy information about studies involving animals; ARRIVE guidelines recommended for reporting animal research

Laboratory animals	The study involved Caenorhabditis elegans day one adult hermaphrodites of various genotypes listed in the Supplementary Data 3. For lifespan, hermaphrodites were analysed from day 1 adulthood until death.
Wild animals	No wild animals were used in this study.
Field-collected samples	No field-collected samples were used in this study.
Ethics oversight	No ethical approval was required since the experiments used solely the invertebrate C. elegans.<br>We followed the guidelines of the German 'Gentechnik-Gesetz' which specifies the work with Genetically Modified Organisms (GMO). Work generated in this study using genetically modified C. elegans falls into risk group 1 and biological safety level 1 (S1) in accordance with the assessment by the 'Zentrale Kommission für die Biologische Sicherheit (ZKBS)'. This requires the correct project documentation, the precise description all S1 GMOs generated and the correct waste disposal of all GMOs.

Note that full information on the approval of the study protocol must also be provided in the manuscript.
